# Prevalence of Risk Factors in Patients with Postprocedural Ischemic Lesions after Coiling of Very Small Intracranial Aneurysms

**DOI:** 10.3390/jcm13133711

**Published:** 2024-06-26

**Authors:** Snezana Lukic, Nenad Zornic, Nemanja Jovanovic, Lukas Rasulic, Vojin Kovacevic

**Affiliations:** 1 Department of Radiology, Faculty of Medical Sciences, University of Kragujevac, 34000 Kragujevac, Serbia; snezanamlukic@gmail.com; 2 Department of Surgery, Faculty of Medical Sciences, University of Kragujevac, 34000 Kragujevac, Serbia; nenadzornic@gmail.com; 3 Clinic of Anesthesiology, University Clinical Center, 34000 Kragujevac, Serbia; 4 Clinic of Neurosurgery, University Clinical Center, 34000 Kragujevac, Serbia; jovanovich.nemanja@gmail.com; 5 Faculty of Medicine, University of Belgrade, 11000 Belgrade, Serbia; lukas.rasulic@gmail.com; 6 Department for Peripheral Nerve Surgery, Functional Neurosurgery and Pain Management Surgery Clinic for Neurosurgery, Clinical Center of Serbia, 11000 Belgrade, Serbia

**Keywords:** very small intracranial aneurysms, endovascular embolization, brain ischemia

## Abstract

**Background:** Very small intracranial aneurysms, generally considered to be those 3 mm in diameter or smaller, pose particular technical challenges for endovascular surgeons. For this reason, very small aneurysms have been excluded from many relevant studies. The aim of our research was to establish the risk factors for the occurrence of stroke complications after endovascular embolization of ruptured and unruptured small intracranial aneurysms. **Methods:** During the period of 2009–2023, our team performed endovascular embolizations of intracranial aneurysms in 1567 patients across four different centers within the territory of Serbia and Montenegro. Within the total number of patients mentioned, aneurysms of less than 4 mm were treated 185 times, with 119 ruptured and 66 unruptured. **Results:** In the group of 119 patients with ruptured small intracranial aneurysms, 19 (16%) patients had ischemia after the endovascular treatment, 6 (5%) patients had minor neurological deficits, while 13 (10.9%) patients had major neurological deficits, of which 6 (5%) patients died. In the group of 66 patients with unruptured small intracranial aneurysms, 7 (10.6%) patients had ischemia after the endovascular treatment, 5 (7.6%) patients had minor neurological deficits, and 2 (3.03%) had major neurological deficits. Multivariate binary logistic regression showed that the risk factors for the occurrence of ischemia were the patient’s age, smoking and alcohol consumption. The type of endovascular treatment used also had a statistically significant effect on the development of ischemia. **Conclusions:** Understanding the influence of possible risk factors for the occurrence of ischemic insult after embolization of small intracranial aneurysms is of great importance. By recognizing them, periprocedural complications can be reduced to a minimum.

## 1. Introduction

Advances in endovascular techniques, primarily in the form of coiling intracranial aneurysms, have led to them becoming a valid alternative to surgical treatment. However, very small intracranial aneurysms, generally considered to be those 3 mm diameter or smaller, pose particular technical challenges for endovascular surgeons [1]. The small diameter of the aneurysmal sac makes stable microcatheter positioning and coil detachment considerably more difficult to achieve. Given the circumstances, in a large number of cases, this increases the risk of procedural aneurysmal rupture, as well as other significant complications [2].

For these reasons, very small aneurysms were excluded from the landmark study by Viñuela et al. [3], a study that led to the approval of the Guglielmi detachable coils in the United States, and from the International Subarachnoid Aneurysm Trial (ISAT) [4]. The International Subarachnoid Aneurysm Trial acknowledged the greater efficacy of endovascular treatment of ruptured aneurysms compared to clipping. But in this trial, very small aneurysms (≤3 mm) were excluded, and the conclusion that coiling is the method of choice is not applicable to these very small aneurysms [5].

In particular, the higher chance of procedural rupture in recently ruptured aneurysms is considered a drawback, and surgery for these very small aneurysms is often advocated. Although the chance of procedural rupture is higher in very small aneurysms, its effect on the outcome is unknown, with only a few reports focusing on the outcome of coiling of the very small aneurysms available [6].

Intracranial aneurysm (IA) rupture is a very common etiologic factor of SAH [7], and IA rupture is among the main reasons for stroke-related severe morbidity and mortality in the United States [8]. Further prognosis for the patient’s outcome after SAH depends on the occurrence of complications such as vasospasm, hydrocephalus, and delayed ischemic deficit, as well as clinical presentation at the time of admission and many other factors [9].

Although, nowadays, they are often diagnosed accidentally, a small percentage of IA can rupture and have devastating health consequences. The top priority regarding IA therapies is to prevent future IA ruptures that could trigger SAH and its associated severe morbidities and potential mortality [9]. Two well-established treatment options currently available for patients presenting with an IA include surgical clipping and endovascular coiling [9,10]. The purpose of both treatments is to exclude the aneurysm from intracranial circulation without occluding the normal vessels [9]. While surgical clipping is less expensive than endovascular coiling [11], endovascular coiling has exhibited a better safety profile in many studies compared to open surgery [12,13]. Furthermore, comorbid conditions that are prevalent in elderly patients, including ischemic stroke, may be a contraindication for surgical clipping, whereas endovascular coiling utilizes thrombogenic coils potentially increasing the risk of ischemic stroke [14]. Our hypothesis was that the rate of ischemic stroke could vary depending on the type of endovascular treatment conducted, independent of patient- and hospital-level characteristics. We also hypothesized that an ischemic stroke diagnosis may modify the relationship between treatment selection and in-hospital outcomes [15].

The aim of our research was to establish risk factors for the occurrence of stroke complications after endovascular embolization of ruptured and unruptured small intracranial aneurysms.

## 2. Material and Methods

During the period of 2009–2023, our team performed endovascular embolizations, as a method of treating intracranial aneurysms, in 1567 patients in 4 different centers on the territory of Serbia and Montenegro. Within the total number of patients mentioned, aneurysms of less than 4 mm were treated 185 times, with 119 ruptured and 66 unruptured (Table 1).

The study focuses retrospectively on a sample of patients who had an intracranial saccular aneurysm of less than 4 mm, ruptured or unruptured, and in whom endovascular embolization was performed as a treatment method. The exclusion criteria were patients diagnosed with multiple aneurysms and/or arteriovenous malformations, occluded blood vessels, an implanted covered stent, as well as with dissecting aneurysms. The study was approved by our institutional review board. All patients or their relatives provided written informed consent during hospitalization.

The factors we analyzed were as follows: age, gender, smoking, alcohol consumption, diabetes mellitus, hypercholesterolemia, hyperlipidemia, hereditary predisposition, previous ischemic insults, aneurysm localization, as well as endovascular treatment modalities (standalone coiling, coiling assisted by a stent, a flow-diverter stent).

All the patients who had neurological complications after the procedure of endovascular embolization of the intracranial aneurysm were classified into the two complication groups. Minor neurological complications were characterized as those that resolved within 7 days, and major neurological complications were those that persisted after 7 days, including fatal outcomes. Adverse events such as transient neurological deficit (hemiparesis, dysphasia) were characterized as minor neurological complications.

### 2.1. Endovascular Treatment

Endovascular embolization of the unruptured intracranial aneurysms was performed in patients who were previously presented to a board of doctors consisting of the following: a neurosurgeon, interventional neuroradiologist, neurologist, and anesthesiologist. Patients with ruptured aneurysms were treated as an emergency in coordination with the neurosurgeon and anesthesiologist. Patients with ruptured aneurysms received preprocedural nimodipine at a dose of 60 mg/24 h to prevent spasms.

Endovascular treatment was always performed under general anesthesia with the systemic administration of heparin and nimodipine. Patients were treated with coiling, stent-assisted coiling, or flow-diverter therapy depending on the morphological features of the aneurysm.

After the procedure was performed, patients with ruptured intracranial aneurysms were treated with nimodipine for the next three weeks—60 mg/24 h in the first week as a systemic application, 40 mg/24 h oral use of the drug during the second week, and 20 mg/24 h in the third week.

If stent-assisted coiling or flow-diverter therapy were planned for the patients presenting with unruptured IAs, 100 mg/day aspirin and 75 mg/day clopidogrel were prescribed for at least 5 days before the procedure. Systemic intravenous heparin was then administered to the patients undergoing the endovascular procedure to maintain an activated clotting time of between 250 and 300 s, to prevent embolic events. After the procedure, all the patients treated with the conventional stent were given 75 mg/day of clopidogrel during a period of 6 weeks, and then 100 mg/day aspirin for 6 months, while patients treated with the flow diverter were given 75 mg/day clopidogrel for 3 months and 100 mg/day aspirin thereafter.

### 2.2. Statistical Analysis

Descriptive statistics, standard deviation, chi-squared tests, and multivariate binary logistic regression were applied. Statistical analyses were performed using the statistical program SPSS 22.0.

## 3. Results

In a group of 119 patients with a ruptured small intracranial aneurysm, 19 (16%) patients developed ischemia after endovascular treatment. Among them, 6 (5%) patients had a minor neurological deficit, and 13 (10.9%) patients had a major neurological deficit. Among patients with a major deficit, death was recorded in 6 (5%) patients, of which 4 had a diagnosed ACM aneurysm. In three patients, the direct cause of death could be attributed to ischemic/hemorrhagic stroke, and in the other three, a series of post-procedural complications supported by comorbidities led to the fatal outcome.

For the clinical grading of the patients after the rupture of a small intracranial aneurysm, the Hunt–Hess (HH) scale was used and the following distribution was recorded: stage HH1—65 (54.6%), HH2—25 (21.0%), HH3—15 (12.6%), HH4—14 (11.8%).

In the group of 66 patients with unruptured small intracranial aneurysms, 7 (10.6%) patients had ischemia after endovascular treatment, 5 (7.6%) patients had a minor neurological deficit, and 2 (3.03%) had a major neurological deficit.

Using the chi-squared test, no correlation was observed between gender and the occurrence of ischemia (*p* = 1.000). Also, there was no correlation between the localization of the small ruptured intracranial aneurysm and ischemia as a complication after the endovascular treatment (*p* = 0.215).

The type of endovascular treatment used had a statistically significant effect on the development of ischemia (*p* = 0.002). The frequency of ischemia in relation to the implemented modality (coil + stent, standalone coil, flow-diverter stent) was 9.5% vs. 9.8% vs. 38.5%, respectively (Table 2).

Multivariate binary logistic regression showed that the occurrence of ischemia depends on the patient’s age (*p* = 0.013), smoking (*p* = 0.001), and alcohol consumption (*p* = 0.001). Age and smoking are independent predictors of ischemia. Each year of age increases the risk of ischemia by 13.4% (Odds ratio = 1.134 (1.027–1.253); Cut-off—54.5 years). Each year of smoking increases the risk by 0.4% (Odds ratio = 1.004 (1.002–1.006)). Hypertension and hypercholesterolemia were not statistically significant for the development of ischemic insult after the endovascular treatment of small ruptured intracranial aneurysms (Table 3).

We also examined the influence of previously diagnosed diabetes mellitus on the development of ischemia. The chi-squared test indicates that the correlation and dependence of the occurrence of ischemic insult after the endovascular treatment of a ruptured small intracranial aneurysm in patients with verified diabetes mellitus (*p* = 0.003) is 9.9% vs. 35.7% (Table 4).

Complications during endovascular treatment are shown in the Table 5. In six patients, mechanical thrombectomy was performed up to the level of the M2 segment of the MCA, as well as up to the A2 segment of the ACA.

## 4. Discussion

Periprocedural ischemic stroke could be a devastating complication of IA endovascular treatment and is a major cause of morbidity or even mortality [16]. In our presented study, we tried to comprehensively evaluate the potential risk factors associated with periprocedural ischemic stroke after the endovascular treatment of intracranial aneurysms.

Although the endovascular treatment of small intracranial aneurysms has become common, it is still an extremely demanding procedure, necessitating the adequate medical preparation of the patient, primarily due to the prevention of vasospasms of the major cerebral arteries. The choice of the treatment modality and the type of embolization material, as well as the implementation of a meticulous operative technique, are also of the great importance for the final treatment outcome.

Treatment modality in our study had significant impact on the development of periprocedural ischemic stroke after the endovascular treatment. Compared with coiling treatment alone, the risk of ischemic events was particularly high after stent-assisted coiling or flow-diverter deployment, which may be due to the thrombogenicity of the intra-arterial devices, longer procedure times, and the complexity of the procedures. Other studies have also obtained results similar to ours [17].

Small aneurysms tend to have a thin and fragile wall as well as restricted space for the insertion of microcatheter and coils, and they have been reported to have higher rates of procedure-related rupture. In a meta-analysis conducted by Brinjikji et al., the incidence of procedural rupture rate among the published studies was 8% [18]. In our study, there were 2.5% reruptures during the embolization of ruptured aneurysms.

In the group of ruptured small intracranial aneurysms, age was the dominant risk factor for the development of an ischemic lesion. After 54.5 years of age, each subsequent year increases the risk of ischemic stroke by 13.4%. The next important risk factor was smoking because each year of smoking increases the risk by 0.4% of the occurrence of ischemia after endovascular treatment.

Periprocedural ischemic strokes usually result from embolic events during the endovascular treatment of intracranial aneurysms, such as the stent wall thrombus, original thrombus, or a fresh clot migrating distally during the procedure [19]. Since platelets are crucial for thrombus formation, inhibition of platelet reactivity may reduce the occurrence of ischemic complications, especially during a treatment option that includes stent placement [20]. Because of that, dual antiplatelet therapy (aspirin and clopidogrel) is widely accepted as the standard protocol to decrease the risk of ischemic events for intracranial aneurysms treated with stents [21].

Diabetes mellitus also significantly increased the risk of post-procedural ischemia development, as well as the presence of excessive alcohol consumption within the patient’s medical history. Chronic hyperglycemia in diabetes promotes “accelerated atherosclerosis“ through the induction of endothelial damage and cellular dysfunction. These two complementary diseases affect the vascular system and, therefore, the risk of developing cardio- and cerebrovascular events becomes evident. Studies have shown that cerebrovascular complications make diabetic patients two to six times more susceptible to an ischemic stroke, and this risk is even more present in younger patients with hypertension and complications in other vascular beds [22].

Our statistical analyses did not verify the significance of the impact of aneurysm localization on the development of ischemia. However, the vast majority of deaths were recorded in the group of patients diagnosed with a ruptured ACM aneurysm (4/6, 66.7%). This result was expected due to the topographic importance of the cerebral vascular territory supplied by the middle cerebral artery (MCA). A study on a large number of patients [23] found that patients with infarcts in the anterior cerebral artery (ACA) territory have a significantly better prognosis as shown by the in-hospital mortality rate in comparison with MCA infarctions (7.8% vs. 17.3%).

Our results also showed that patients with unruptured small intracranial aneurysms were significantly younger compared to the group of patients diagnosed with ruptured aneurysms. Unruptured aneurysms are mostly diagnosed as an incidental finding during neuroradiological examinations. After a follow-up on control MR examinations, due to the change in shape and increase in size, as well as the presence of risk factors, a decision was made to apply endovascular treatment. The significantly lower average age of the patients (48.6 vs. 59.7 years old) could also explain the significantly lower incidence of ischemic lesions in the group of patients who had an unruptured aneurysm.

We are also aware that our study has some limitations. Our analysis was conducted retrospectively and may impair the findings of this study. This may introduce selection bias not only prior to the procedure but also during the postprocedural period as patients who experienced better outcomes may be less likely to attend the follow-up. The lack of certain clinical parameters and data on the course of hospitalization could significantly affect our conclusions regarding periprocedural complications and the final treatment outcomes. Several studies have concluded that variables such as temperature > 38 °C 8 days after SAH, liver disease, and the use of anticonvulsants/early seizures were associated with a poor outcome [24]. A study by a Canadian group of authors especially emphasized the importance of hypertension and liver disease in the development of brain swelling, as well as the negative consequences of seizures in patients with a history of myocardial infarctions and post-admission fevers worsening the neurological outcomes [25]. The aforementioned studies were also retrospective.

However, the strength of our study lies in the large number of patients with treated small aneurysms, who constitute an even more complicated subgroup of patients for the treatment of an already complex disease.

## 5. Conclusions

In our study, age and smoking are the dominant risk factors for the development of ischemia. Patients suffering from diabetes mellitus are also at a higher risk of developing ischemia. Compared with coiling treatment alone and stent-assisted coiling, the risk of ischemic events is significantly higher after flow-diverter deployment.

Understanding the influence of possible risk factors for the occurrence of ischemic insult after embolization of small intracranial aneurysms is of great importance. With adequate pre-procedural preparation of patients, it is possible to reduce the negative effects of these factors and also reduce the rate of treatment complications to an acceptable minimum. Also, by choosing the appropriate technique and embolization materials, endovascular treatment is still the method of choice for treating very small intracranial aneurysms, both ruptured and unruptured.

Future research must aim to prospectively include a wider range of risk factors that can potentially have a significant impact on the development of postprocedural ischemia. Attention should first be directed at the aging population, from which comes a large number of patients who are highly likely to have numerous comorbidities.

## Figures and Tables

**Table 1:** Results from univariate statistical analysis for all variables.

Characteristics	Ruptured Aneurysm Group No 119	Non-Ruptured Aneurysm Group No 66
Age (years)	59.7	48.6
Male	44 (36.9)	22 (33.3)
Female	75 (63.1)	44 (66.7)
Drinking %	45 (37.8)	18 (27.3)
Smoking %	58 (48.7)	31 (26)
Hypertension %	64 (53.8)	29 (43.9)
Hyperlipidemija %	49 (41.2)	28 (42.4)
Genetic predisposition %	25 (21)	19 (28.8)
Shape Regular %	84 (70.6)	24 (36.4)
Irregular %	35 (29.4)	42 (63.6)
Location (%)		
ICA	31 (26.1)	20 (30.3)
MCA	36 (30.2)	24 (36.3)
A.Comm.ant	18 (15.1)	14 (21.2)
PICA	4 (3.4)	0 (0.0)
A.Com.post	8 (6.72)	0 (0.0)
BA	14 (11.8)	6 (9.1)
PCA	3 (1.6)	1 (1.5)
Distal Location	5 (4.2)	1 (1.5)
Treatment therapy %		
Coiling	51 (42.9)	31 (46.9)
Stent-assisted coiling	42 (35.3)	29 (44)
Flow-diverter stent	26 (21.8)	6 (9.1)

**Table 2:** Statistical analysis of the influence of the type of applied endovascular treatment and the occurrence of ischemia.

Treatment Therapy	Ischemia		Total
	0.00	1.00	
Coil + stent	38	4	42
	90.5%	9.5%	100.0%
Coil	46	5	51
	90.2%	9.8%	100.0%
Flow-divert stent	16	10	26
	61.5%	38.5%	100.0%
Total	100	19	119
	84.0%	16.0%	100.0%

**Table 3:** Statistical analysis using multivariate binary regression on the present risk factors.

	B	S.E.	Wald		df	Sig.	Exp (B)	95% C.I. for EXP (B)	
								Lower	Upper
Age	0.126	0.051		6.166	1	0.013	1.134	1.027	1.253
Hypertension	−1.913	1.218		2.469	1	0.116	0.148	0.014	1.605
Diabetes	−0.573	1.001		0.327	1	0.567	0.564	0.079	4.015
Hypercholesterolemia	0.875	1.118		0.614	1	0.433	2.400	0.268	21.453
Smoking	0.004 0.004	0.001 0.001		10.683 10.680	1 1	0.001 0.001	1.004 1.002	1.002 1.001	1.006 1.005
Constant	−7.856	2.460		10.196	1	0.001	0.000		

Variable(s) entered at step 1: age, hypertension, diabetes, hypercholesterolism, smoking and alcohol.

**Table 4:** Correlation and dependence of the occurrence of ischemic insult after endovascular treatment of a small ruptured intracranial aneurysm in patients with verified diabetes mellitus (*p* = 0.003). 9.9% vs. 35.7%.

			Ischemia		Total
			0.00	1.00	
Diabetes	0.00	Count	82	9	91
		% within diabetes	90.1%	9.9%	100.0%
	1.00	Count	18	10	28
		% within diabetes	64.3%	35.7%	100.0%
Total		Count	100	19	119
		% within diabetes	84.0%	16.0%	100.0%

**Table 5:** Procedural complications.

Type of Complication	Group of Ruptured Aneurysms No 119 (%)	Group of Unruptured Aneurysms No 66 (%)
Rerupture	3 (2.5%)	0 (0%)
Coil migration	2 (1.7%)	1 (1.5%)
Migration of the stent	1 (0.84%)	0 (0%)
Distal embolization	5 (4.2%)	2 (3.03%)
Spasm	18 (15.1%)	2 (3.03%)
Total	29 (24.4%)	8 (12%)

## Data Availability

The raw data supporting the conclusions of this article will be made available by the authors on request.

## Abbreviations

ACA	anterior cerebral artery
BA	basilar artery
C	Celsius
EE	endovascular embolization
HH	Hunt–Hess
IA	intracranial aneurysm
ICA	internal carotid artery
MCA	midle cerebral artery
PCA	posterior cerebral artery
PICA	posterior inferior cerebellar artery
SAH	spontaneous subarachnoid hemorrhage
